# Barcoding snakeheads (Teleostei, Channidae) revisited: Discovering greater species diversity and resolving perpetuated taxonomic confusions

**DOI:** 10.1371/journal.pone.0184017

**Published:** 2017-09-20

**Authors:** Cecilia Conte-Grand, Ralf Britz, Neelesh Dahanukar, Rajeev Raghavan, Rohan Pethiyagoda, Heok Hui Tan, Renny K. Hadiaty, Norsham S. Yaakob, Lukas Rüber

**Affiliations:** 1 Naturhistorisches Museum der Burgergemeinde Bern, Bern, Switzerland; 2 Department of Life Sciences, Natural History Museum, London, United Kingdom; 3 Indian Institute of Science Education and Research, Pashan, Pune, Maharashtra, India; 4 Systematics, Ecology & Conservation Laboratory, Zoo Outreach Organization, Saravanampatti, Coimbatore, Tamil Nadu, India; 5 Department of Fisheries Resource Management, Kerala University of Fisheries and Ocean Studies, Kochi, Kerala, India; 6 Ichthyology Section, Australian Museum, Sydney, Australia; 7 Lee Kong Chian Natural History Museum, National University of Singapore, Singapore, Singapore; 8 Museum Zoologicum Bogoriense, Research Center for Biology, Indonesian Institute of Sciences, Cibinong, Indonesia; 9 Forest Research Institute Malaysia, Kepong, Kuala Lumpur, Malaysia; 10 Institute of Ecology and Evolution, University of Bern, Bern, Switzerland; University of Minnesota, UNITED STATES

## Abstract

Snakehead fishes of the family Channidae are predatory freshwater teleosts from Africa and Asia comprising 38 valid species. Snakeheads are important food fishes (aquaculture, live food trade) and have been introduced widely with several species becoming highly invasive. A channid barcode library was recently assembled by Serrao and co-workers to better detect and identify potential and established invasive snakehead species outside their native range. Comparing our own recent phylogenetic results of this taxonomically confusing group with those previously reported revealed several inconsistencies that prompted us to expand and improve on previous studies. By generating 343 novel snakehead coxI sequences and combining them with an additional 434 coxI sequences from GenBank we highlight several problems with previous efforts towards the assembly of a snakehead reference barcode library. We found that 16.3% of the channid coxI sequences deposited in GenBank are based on misidentifications. With the inclusion of our own data we were, however, able to solve these cases of perpetuated taxonomic confusion. Different species delimitation approaches we employed (BIN, GMYC, and PTP) were congruent in suggesting a potentially much higher species diversity within snakeheads than currently recognized. In total, 90 BINs were recovered and within a total of 15 currently recognized species multiple BINs were identified. This higher species diversity is mostly due to either the incorporation of undescribed, narrow range, endemics from the Eastern Himalaya biodiversity hotspot or the incorporation of several widespread species characterized by deep genetic splits between geographically well-defined lineages. In the latter case, over-lumping in the past has deflated the actual species numbers. Further integrative approaches are clearly needed for providing a better taxonomic understanding of snakehead diversity, new species descriptions and taxonomic revisions of the group.

## Introduction

Species identification and delimitation play a vital role in our understanding of the diversity of life. Despite calls for integrative approaches in biodiversity studies [1,2], traditional morphology based approaches are being rapidly supplanted by approaches that solely rely on DNA-based data. While studies using multi-locus data are clearly superior in identifying species boundaries [3,4], single-locus data dominate DNA taxonomy, not least because of the increased popularity of DNA barcodes in biodiversity research [5]. As a consequence the last ten years have seen a rapid proliferation of scalable molecular approaches for automatic species delimitation based on single-locus data e.g. [6–11]; but see [12] for a critical view on the utility of single-locus approaches. These analytical approaches can be classified into three main groups [11]: clustering, tree-based and character-based methods, with the former two approaches clearly dominating the burgeoning field of molecular species delimitation. While clustering methods use different algorithms to detect discontinuities in genetic distance matrices, gene trees are used as the basis in tree-based methods. Among the most popular clustering methods are the Automatic Barcode Gap Discovery (ABGD, [13]) and the Refined Single Linkage (RESL) / Barcode Index Number (BIN), methods [14], hereafter referred to as BIN only. They are consistent in identifying the presence of a 'barcoding gap', the discontinuity between intra- and interspecific sequence divergences, but are prone to fail when these two classes of pairwise genetic distances overlap [15]. Widely used tree-based approaches on the other hand are for example the Generalized Mixed Yule Coalescent (GMYC, [7,16]) and Poisson Tree Processes (PTP, [17]) methods. Several recent studies have looked at different aspects of species delimitation and their effect on inferred species diversity based on: the different methods used [6,11,14]; the phylogenetic reconstruction methods used [18,19]; the presence of singletons and various degrees of incomplete sampling in the data set [9,18,20,21]; the geographic scale of taxon sampling [22]; and dispersal ability and migration rates and their impact on the formation of discrete genetic clusters [12,23].

Single-locus based species delimitation approaches are particularly useful in taxonomic groups that are understudied or characterized by taxonomic difficulties and confusion. One such group suffering from these issues are the snakehead fishes of the family Channidae, a group of predatory freshwater teleosts that comprises two genera: *Channa*, with 35 valid species distributed from the Middle East to eastern Asia and *Parachanna* with three species in Central and West Africa and the Nile. What has made this small number of only 38 species taxonomically notorious is due to several factors: 1) a large number of synonyms stemming from the early periods of ichthyological exploration when large scale revisions were lacking and species were described based on small numbers of specimens, 2) striking changes in colour pattern throughout ontogeny, often involving different larval, juvenile, sub-adult and adult patterns and 3) periods where splitters and lumpers alternated and interpreted species complexes in very different ways leading to a confusion about the actual number of valid species. Adding to this already unsatisfactory condition have been a large number of species descriptions in the last two decades, some of which have not properly looked at previous published works with the necessary rigour. This is particularly problematic, as snakeheads are important food fishes (aquaculture, live food trade), some of which have been introduced widely and have developed into invasive species [24,25]. Others are utilized commercially in the ornamental fish trade [26] with one species, *Channa barca*, fetching prices of one to several thousand dollars per piece.

Several molecular phylogenetic studies in the recent past have addressed channid intrarelationships (e.g. [27–29] or have explored channid species diversity by means of DNA barcodes (e.g. [30–32]). In order to provide better tools for the detection and identification of potential and established invasive snakehead species outside their native range, Serrao *et al*. [32] assembled the largest channid DNA barcode library thus far representing 25 of the 38 valid species. Among the 250 individuals in their study (121 newly generated cytochrome *c* oxidase I (coxI) sequences and 129 from GenBank) they identified a total of 49 haplogroups or BINs, 19 of which were represented by single specimens. When comparing the barcode results of [32] with those of our own ongoing investigations into the molecular phylogenetics of snakeheads we discovered several inconsistencies, prompting the present study. For example, the presence of an unidentified *Channa* species from Sumatra in their analysis, which is resolved as sistergroup to all remaining *Channa* species, raised some questions. To scrutinize and critically check the channid DNA barcode library presented by [32], we undertook a comprehensive barcoding study based on 777 coxI sequences, including 343 coxI sequences generated specifically for this study from DNA samples of specimens identified by taxonomic experts of the family Channidae (RB, HHT), complemented by 434 coxI sequences from GenBank.

## Material and methods

### Ethics statement

Fieldwork in Peninsular Malaysia and Sarawak was conducted under permits issued by the Economic Planning Unit, Prime Minister’s Department, Malaysia (UPE 40/200/19/2417 and UPE 40/200/19/2534) and the Forest Department Sarawak (NCCD.970.4.4[V]-43) and fieldwork in Sumatra and Borneo was conducted under permits issued by the Indonesian Institute of Sciences (LIPI) and the Kementerian Negara Riset dan Teknology (RISTEK; 1/ TKPIPA/FRP/SM/I/2011 and 3/TKPIPA/FRP/SM/III/2012) in collaboration with the Museum Zoologicum Bogoriense. Permits for collecting in Myanmar and Vietnam were issued by the Department of Fisheries, Ministry of Livestock Breeding & Fisheries, Yangon and the Vietnam National Museum of Nature, respectively. Samples from India were collected from non-protected areas for which the permissions were not required as none of the *Channa* species fall under the Indian Wildlife Protection act. No ethical approval was required for this study because no experimentation or manipulations were carried out and there is no relevant legislation. In the field, fish were either caught using dip nets, push nets or seines or were obtained from local fish markets. Additional specimens were obtained through the aquarium trade in Germany, Singapore, and the UK. All samples from the aquarium trade were obtained before the Nagoya Protocol on Access and Benefit-sharing was implemented on 12 October 2014. Details and source of samples are provided in S1 Table. Immediately upon capture in the field or after purchase from the aquarium trade in Europe, specimens were killed by an overdose of anaesthesia using MS222 following guidelines by the American Society of Ichthyologists and Herpetologists (ASIH) (http://www.asih.org/pubs/; issued 2013) and sampled. In the markets, samples were taken from dead specimens. Muscle tissue samples or fin clips were subsequently stored in 100% ethanol and voucher specimens were then preserved in either 4% formalin or 75% ethanol.

### Taxon sampling, DNA extraction, PCR amplification, sequencing, and alignments

To extend the existing channid DNA barcode library, we newly generated coxI nucleotide sequences from 343 individuals, not previously used in any molecular analysis. Total genomic DNA was extracted from muscle tissues or fin clips preserved in 100% ethanol and stored at -80°C using the DNeasy Blood and Tissue Kit (Qiagen) following manufacturer’s instructions. Some extractions were conducted on a QIAcube robotic workstation. Partial coxI fragments were PCR amplified in 25 μl reactions using the Promega Green Master Mix (Promega) following the manufacturer’s protocol and 1.5 μl template DNA using the primers FishF1cox1 or FishF2cox1 and FishR2cox1 [33]. PCR condition were: 3 minutes at 94°C; 35 cycles of 30 seconds at 95°C, 30 seconds at 52°C and 1 minute at 72°C; 7 minutes at 72°C and holding at 10°C. Alternatively, for difficult templates coxI fragments were PCR amplified in 25 μl using the Qiagen Multiplex PCR Mix and using the PCR conditions according to the manufacturer's protocol. PCR products were checked visually by electrophoresis on a 1.5% agarose gel. PCR cleanup and Sanger sequencing for both strands using the PCR primers were conduct by LGC Genomics, Berlin.

For the coxI sequences generated in India, the following protocols were used. Gills were harvested from fresh specimen and were preserved in 100% ethanol. DNA was extracted using QIAamp DNA Mini Kit (Qiagen) following manufacturer’s instructions. Partial COI fragments were PCR amplified using primers FishF1cox1 and FishR1cox1 [33]. PCR reaction was performed in a 25μl reaction volume containing 5μl of template DNA (~200ng), 12.5μl of Promega 2X PCR Master Mix, 1μl of each primer and 5.5 μl nuclease free water. The thermal profile was 10 minutes at 95°C, and 35 cycles of 1 minute at 94°C, 1 minute at 56°C and 2 minutes at 72°C, followed by extension of 10 minutes at 72°C. Amplified DNA fragments were purified using the Wizard SV Gel and PCR clean-up system (Promega). Sanger sequencing was conducted by 1st BASE, Axil Scientific Pte Ltd, Singapore.

Chromatogram traces/raw reads were edited and assembled into contigs using Geneious v8.1.3 [34]. In addition to the 343 channid coxI sequences generated for this study, we also added to our data set all available coxI sequences stored as belonging to the family Channidae in GenBank. We retrieved a total of 497 sequences from GenBank (www.ncbi.nlm.nih.gov, accessed March 31, 2015) of which 434 were retained after closer inspection (see Results for more details). The coxI sequences of 777 channid specimens and one outgroup (*Nandus nandus*, GeneBank accession number JQ713845) based on [35] were aligned with MAFFT v7.017 ([36]) as implemented in Geneious v8.1.3 [34] using the default settings. The alignment was checked for frameshifts and premature stop codons. This data set will be referred to as the 778 taxa data set throughout the manuscript. Details of all 777 channid specimens used in this study such as voucher number, locality information, GPS coordinates, and GenBank accession numbers are provided in S1 Table. Some of the analyses (see below) were based on a reduced data set (423 taxa data set) containing only unique channid haplotypes (n = 422; see S1 Table) plus the outgroup.

### Data analyses

The final alignment was subjected to phylogenetic analyses using neighbour joining (NJ), maximum likelihood (ML) and Bayesian Inference (BI). The NJ analyses using HKY distances were conducted in PAUP* v4.0a147 [37]. Alternative pairwise distances (GTR, K2P) for the NJ analyses were explored and resulted in comparable phylogenetic hypotheses and hence are not shown. PartitionFinder 1.0.1 [38] was used to assess the optimal partitioning for subsequent ML analyses using RAxML v8.2.X [39], and BI analyses using BEAST v1.8.0 [40] and substitution model scheme (for subsequent BEAST analysis) for the coxI alignment using three potential partitions as input (coxI first, second and third codon positions). PartitionFinder was run separately for the RAxML and BEAST analyses with the following settings: models = raxml (for the subsequent RAxML analyses) or beast (for the subsequent BEAST analyses); model_selection = BIC (all analyses); search = greedy (all analyses). Both, ML (RAxML) and BI (BEAST) analyses were conducted on the reduced 423 taxa data set only. RAxML was used to conduct the ML analyses by implementing the GTRGAMMA model for all partitions as identified by PartitionFinder (see RAxML manual for justification) using option -f a which conducts a rapid bootstrap analysis (500 pseudoreplicates) and searches for the best-scoring ML tree by computing ten distinct ML trees starting from ten distinct randomized maximum-parsimony starting trees in a single program run. For the ML analysis we enforced a topological constraint (*Parachanna* and *Channa* are sister groups). Each analysis was run three times with different starting seeds. Results for these three independent runs were highly congruent and thus only the run with the highest log-likelihood score was retained.

Since some of the subsequent species delimitation methods (see below) required an ultrametric tree, we conducted a BI analysis using BEAST v1.8.0 using an uncorrelated lognormal relaxed molecular clock implementing a coalescent tree prior. According to the results from PartitionFinder (see Results) we used three partitions using the option unlink substitution model. We further used the option link clock model using one model for the entire coxI and we linked all three tree models. We changed the following priors from their default value: clock rate (usld.mean) for the three genes were changed to Gamma (1, 1), initial = 1; and all p substitution parameters (GTR substitution parameters) were changed from Gamma to InverseGamma. One lognormal calibration prior from the fossil record [41] was used: time of most recent common ancestor of *Parachanna* stem (offset 33.0; 37.0 Ma 95% soft upper bound; log mean = 0.1; log stdev = 0.8). The Markov chain Monte Carlo (MCMC) chain was run two times for 10^8^ generations, sampling every 20,000 generations. The resulting tree and log files were combined in LogCombiner v1.8.0 [40] using a conservative burnin of 10%. Chain convergence and effective sample size (ESS; all ESS > 200) were verified using Tracer v1.6 [40] and the resulting ultrametric tree, the maximum clade credibility tree, calculated from the BEAST posterior distribution with TreeAnnotator v1.8.0 [40] was visualized and exported for subsequent analyses using FigTree v1.4.2 (http://tree.bio.ed.ac.uk/software/figtree/).

### Species delimitation analyses

We chose three commonly used methods for single-locus DNA-based species delimitation: BIN, GMYC and PTP. Firstly, we employed the BIN analysis that at its core uses the RESL algorithm [14], a clustering method that produces a matrix of pairwise distances (uncorrected p-distances) comparing all barcode sequences to a reference database and then clustering the unidentified sequence based on a pre-assigned p-distance threshold, thereby providing unique BIN numbers for each Operational Taxonomic Unit (OTU). Contrary to the assertion by [11] there is still no public release of a stand-alone version of RESL to conduct BIN analyses. Hence, we had to use the standard BIN assignment available through the ID tool in BOLD (http://www.boldsystems.org/bin) that is based on all barcode sequences on BOLD, a more inclusive dataset, and thus the results are not exactly comparable to those obtained with the GMYC and PTP method.

We used the BOLD identification tool (accessed March 31, 2016) to assign all the 777 channid coxI sequences in this study to existing BIN numbers. These sequences were assigned to either the channid BINs already reported by [32] or to new public and non-public BINs reported in BOLD for snakeheads (see Results for more detail). Sequences that could not be assigned to existing BINs were regarded as potentially belonging to new BINs.

Secondly, we used two commonly used tree-based species delimitation methods, well suited for single-locus data, PTP and GMYC. While PTP can use both ultrametric and non-ultrametric trees as input, GMYC only uses ultrametric trees and thus the former method does not require time-consuming branch smoothing steps. For our analyses we largely followed [19] who recommend the simultaneous use of the PTP method based on model-based ML gene trees and GMYC approaches based on ultrametric BEAST trees for obtaining species hypotheses. We performed both, a PTP analysis in a ML framework and a bPTP analysis in a Bayesian framework using Phyton scripts available at http://sco.h-its.org/exelixis/web/software/PTP/index.html. Both methods model the speciation branching patterns in terms of substitution numbers [17]. Our PTP analysis was based on the ML tree from our RAxML analysis as input, whereas the bPTP analysis was conducted on 100 randomly chosen trees from the RAxML boostrap analysis with a MCMC chain length of 500,000 generations and sampling every 250th generation and with a burn-in of 10%. As a second group of tree-based methods we used GMYC [7,8,16] with single- and multiple-threshold features and with the Bayesian implementation (bGMYC, [42]). These approaches identify independent lineages by detecting a threshold value at the transition from coalescent to speciation branching patterns. In turn, they require time calibrated phylogenetic trees with branch lengths representing time. The GMYC single- and multiple-threshold algorithms were employed using the R-package *splits* [43] based on the maximum clade credibility tree. For the bGMYC analysis we used the R-packages *bGMCY* [42], *phangorn* [44] and *ape* [45] using 100 randomly chosen ultrametric trees obtained from the BEAST posterior distribution as input. The settings for the bGMYC analysis were: MCMC chain length = 500,000 generations, sampling every 100th generations and a 10% burn-in, t2 (upper threshold parameter) = 160 and starting value = 90.

Although, a 'global' barcoding gap might not exist in most lineages due to extensive overlap between intra- and interspecific distances caused by variation in coalescent depth, the identification of a 'local' barcoding gap is more useful for species identification and delimitation purposes. To this end and following [46] we plotted for each individual the distance to the furthest conspecific individual against the distance to the nearest non-conspecific individual. Here, the 1:1 slope demarcates the areas 'local' and 'no local' barcoding gap. We used two different taxonomic groupings for this analyses, species plus intraspecific clades and BIN assignments. The distance calculations and dotplots were conducted with R scripts from the spider package [47] and R scripts provided by R. Collins.

## Results

### Summary of molecular data and phylogenetic analyses

For this study we newly determined 343 channid coxI sequences and deposited them in GenBank under accession numbers MF462263- MF462283 and MF496660—MF496981 (S1 Table). In addition, we downloaded 497 channid coxI sequences from GenBank, but had to discard 59 because they did not cover the coxI fragment used for DNA barcoding of fishes. An additional three coxI sequences (accession numbers JF900369, JQ667513, JX983250) were excluded from the final alignment due to poor sequence quality (e.g. extra base pairs at the 5' and 3' end of the sequences leading to frame shifts). And finally, one additional sequence (accession number KJ937355) was highly divergent from the other channid coxI sequences upon visual inspection of the preliminary alignment. A megablast search of this *Channa* sp. (KJ937355), sister group to all remaining *Channa* species in [32], revealed a 94–95% identity with three individuals of the cyprinid species *Rasbora trilineata* (accession numbers KC456379, EF452883 and KM200714) and was thus excluded since it almost certainly represents a case of a sample mix-up.

The resulting 434 channid coxI sequences downloaded from GenBank we retained included: a) 120 out of the 121 sequences generated by [32] (sequence KJ937355 was excluded, see above). b) 124 of the 129 channid coxI sequences downloaded from GenBank by [32]. We did not include five sequences (accession numbers JX978723, JX978725, KC310861, and NC_015191) representing complete mitochondrial genomes of the species *Channa argus* and *Channa maculata* because they did not show up initially during our GenBank searches. c) an additional 190 channid coxI sequences from GenBank not previously used by [32]. The final alignment of 777 channids plus one outgroup was 654 bp long and is deposited in Dryad (doi:10.5061/dryad.7h0g6).

**Fig 1:**
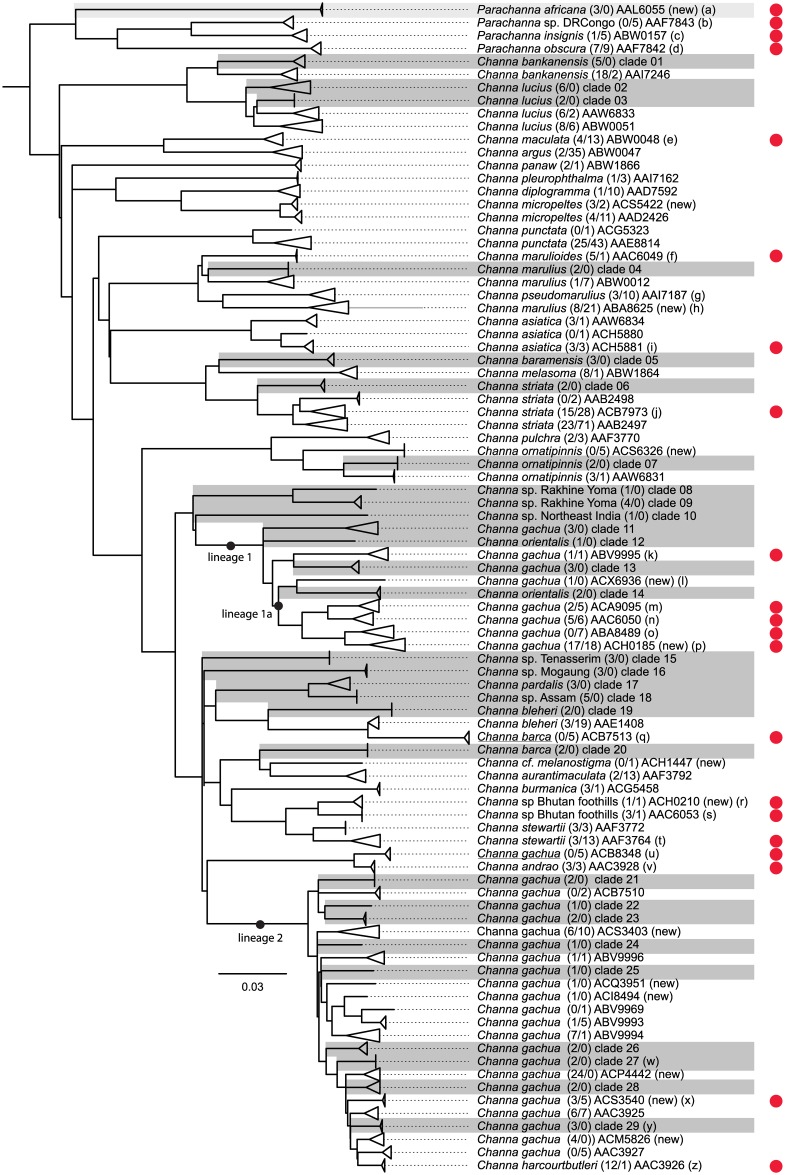
Distance tree of snakehead barcodes. Neighbour joining tree based on HKY distances of 777 channid coxI sequences. Individuals have been collapsed into 61 BINs and 29 potential new BINs (indicated as clades 1–29 and highlighted in dark grey); see Table 1. The *Parachanna africana* clade is highlighted in light grey. Numbers in brackets behind species names refer to number of individuals from this study / number of individuals from GenBank, followed by BIN or clade designation. BINs from BOLD not previously reported by [32] are indicated in brackets with "new" (BOLD accessed March 31, 2016). Red dots indicate clades containing misidentified or incomplete identified specimens; see Table 2 and the following comments: (a) *Protopterus annectens* BIN containing five lungfish specimens, (b) three *Pa*. *africana* and two *Pa*. *obscura* included, (c) one *Pa*. sp included, (d) two *Pa*. *insignis* included, (e) nine *Channa* sp. included, (f) one *C*. cf. *marulius* included, (g) ten *C*. *marulius* with accession numbers EU342199-EU342200, HM117192-HM117196, KJ937341, KJ937348, KJ937388 included. These might be *C*. *pseuomarulius*, but were not counted as misidentifications since this species was only recently revalidated by Britz *et al*. [48], (h) includes an exceptionally long branch for a single specimen (accession number JX983243 shown as a grey bar) probably due to sequencing errors, (i) one *C*. cf. *nox* included, (j) five *C*. *marulius* included, (k) one *C*. *orientalis* included, (l) BIN number is for unpublished *C*. *orientalis* in BOLD, (m) five *C*. *orientalis* included, (n) six *C*. *orientalis* included, (o) six *C*. *orientalis* included, (p) 17 *C*. *orientalis* included, (q) misidentification should be *C*. *bleheri* see Table 2, (r) one C. *stewartii* included, (s) one *C*. cf. *stewartii* included, (t) five *C*. *barca* included, (u) misidentification should be *C*. *andrao* see Table 2, (v) three *C*. *gachua* included, (w) BOLD ID showed 97.39% similarity with ACS3540, (x) five *C*. *stewartii* included, (y) BOLD ID showed 97.98% similarity with AAC3925, (z) one *C*. *gachua* included. For clades composed of misidentified specimens only, the original species designation was kept and these clades are underlined (ACB7513 and ACB8348).

**Fig 2:**
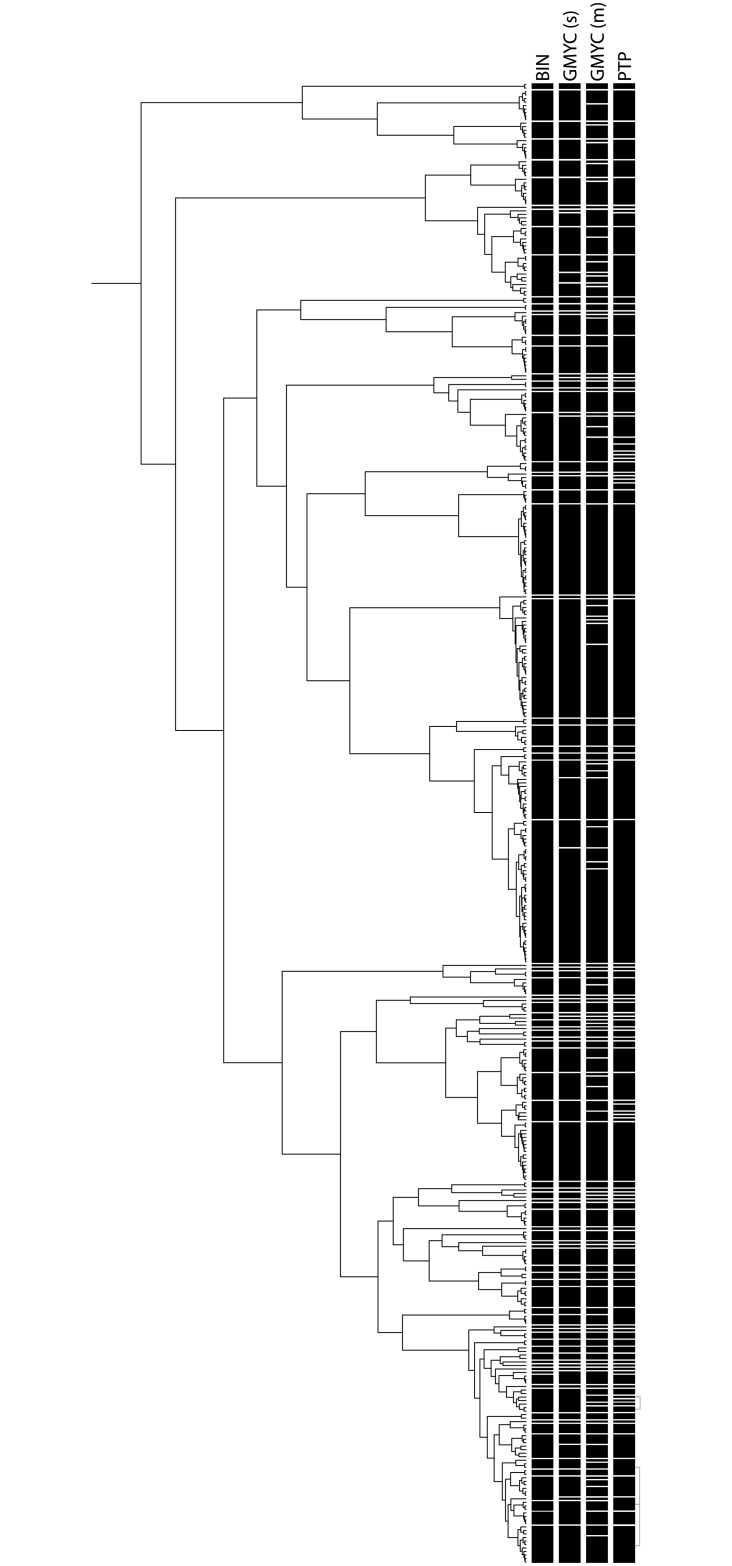
Snakehead chronogram. BEAST analysis using a coalescence prior. Species delimitations based on BIN, GMYC single, GMYC multiple, and PTP thresholds are indicated by black bars. The same figure with species names is given in S4 Fig.

According to the results from PartitionFinder we used three partitions (with GTRGAMMA for each partition, see Material and Methods) for the RAxML analysis and also three partitions (1st codon position = TrNef+I+G, 2nd codon position = HKY+G, 3rd codon position = GTR+G) for the BEAST analysis of the 423 taxa data set. The collapsed NJ tree of the 778 taxa data set is shown in Fig 1 along with the assigned BIN numbers (see below and Table 1 and S1 Table). The uncollapsed NJ tree is shown in S1 Fig and the corresponding 50% majority bootstrap consensus tree is shown in S2 Fig. The ML tree of the 423 taxa data set is shown in S3 Fig, this is the tree that was used for the subsequent PTP analysis. The channid timetree from the BEAST analysis based on the 423 taxa data set that was subsequently used for the GMYC analyses is shown in Fig 2. The major channid clades and subclades were largely congruent across the different analyses.

**Table 1:** Summary of channid species and their BIN and clade assignment following the clade order in Fig 1. Species names according to [32] if different from this study are given, BINs reported by [32] are indicated and those species with multiple BINs in the [32] study and in this study are indicated.

Clade number	Species name this study	Species name Serrao *et al*. [32]	BIN / clade number	BIN Serrao *et al*. (2014)	BIN group Serrao *et al*.	BIN group this study
1	*Parachanna africana*	*Protopterus annectens*^(a)^	AAL6055	no		
2	*Parachanna* sp. DRCongo	*Parachanna africana*	AAF7843	yes		
3	*Parachanna insignis*	n/a	ABW0157	no	1	
4	*Parachanna obscura*	n/a	AAF7842	yes		
5	*Channa bankanensis*	n/a	clade 01	no		1
6	*Channa bankanensis*	n/a	AAI7246	yes		1
7	*Channa lucius*	n/a	clade 02	no		2
8	*Channa lucius*	n/a	clade 03	no		2
9	*Channa lucius*	n/a	AAW6833	yes	2	2
10	*Channa lucius*	n/a	ABW0051	yes	2	2
11	*Channa maculata*	n/a	ABW0048	yes		
12	*Channa argus*	n/a	ABW0047	yes		
13	*Channa panaw*	n/a	ABW1866	yes		
14	*Channa pleurophthalma*	n/a	AAI7162	yes		
15	*Channa diplogramma*	n/a	AAD7592	yes		
16	*Channa micropeltes*	n/a	ACS5422	no		3
17	*Channa micropeltes*	n/a	AAD2426	yes		3
18	*Channa punctata*	n/a	ACG5323	yes	3	4
19	*Channa punctata*	n/a	AAE8814	yes	3	4
20	*Channa marulioides*	*Channa* cf. *marulius*	AAC6049	yes		
21	*Channa marulius*	n/a	clade 04	no		5
22	*Channa marulius*	n/a	ABW0012	yes	4	5
23	*Channa pseudomarulius*	*Channa marulius*	AAI7187	yes	4	
24	*Channa marulius*	n/a	ABA8625	no		5
25	*Channa asiatica*	n/a	AAW6834	yes	5	6
26	*Channa asiatica*	n/a	ACH5880	yes	5	6
27	*Channa asiatica*	n/a	ACH5881	yes	5	6
28	*Channa baramensis*	n/a	clade 05	no		
29	*Channa melasoma*	n/a	ABW1864	yes		
30	*Channa striata*	n/a	clade 06	no		7
31	*Channa striata*	n/a	AAB2498	yes	6	7
32	*Channa striata*	n/a	ACB7973	yes	6	7
33	*Channa striata*	n/a	AAB2497	yes	6	7
34	*Channa pulchra*	n/a	AAF3770	yes		
35	*Channa ornatipinnis*	n/a	ACS6326	no		8
36	*Channa ornatipinnis*	n/a	clade 07	no		8
37	*Channa ornatipinnis*	n/a	AAW6831	yes		8
38	*Channa* sp. Rakhine Yoma	n/a	clade 08	no		9
39	*Channa* sp. Rakhine Yoma	n/a	clade 09	no		9
40	*Channa* sp. Northeast India	n/a	clade 10	no		
41	*Channa gachua*	n/a	clade 11	no		10
42	*Channa orientalis*	n/a	clade 12	no		11
43	*Channa gachua*	*Channa orientalis*	ABV9995	yes	7	10
44	*Channa gachua*	n/a	clade 13	no		10
45	*Channa gachua*	n/a	ACX6936	no		10
46	*Channa orientalis*	n/a	clade 14	no		11
47	*Channa gachua*	*Channa orientalis*	ACA9095	yes	7	10
48	*Channa gachua*	*Channa orientalis*	AAC6050	yes	7	10
49	*Channa gachua*	*Channa orientalis*	ABA8489	yes	7	10
50	*Channa gachua*	n/a	ACH0185	no		10
51	*Channa* sp. Tenasserim	n/a	clade 15	no		
52	*Channa* sp. Mogaung	n/a	clade 16	no		
53	*Channa pardalis*	n/a	clade 17	no		
54	*Channa* sp. Assam	n/a	clade 18	no		
55	*Channa bleheri*	n/a	clade 19	no		12
56	*Channa bleheri*	n/a	AAE1408	yes		12
57	*Channa bleheri*	*Channa barca*	ACB7513	yes		12
58	*Channa barca*	n/a	clade 20	no		12
59	*Channa* cf. *melanostigma*	n/a	ACH1447	no		
60	*Channa aurantimaculata*	n/a	AAF3792	yes		
61	*Channa burmanica*	n/a	ACG5458	yes		
62	*Channa* sp. Bhutan foothills	*Channa stewartii*	ACH0210	no		13
63	*Channa* sp. Bhutan foothills	*Channa* cf. *stewartii*	AAC6053	yes		13
64	*Channa stewartii*	n/a	AAF3772	yes	8	14
65	*Channa stewartii*	n/a	AAF3764	yes	8	14
66	*Channa andrao*	*Channa gachua*	ACB8348	yes	9	15
67	*Channa andrao*	*Channa gachua*	AAC3928	yes	9	15
68	*Channa gachua*	n/a	clade 21	no		10
69	*Channa gachua*	n/a	ACB7510	yes	9	10
70	*Channa gachua*	n/a	clade 22	no		10
71	*Channa gachua*	n/a	clade 23	no		10
72	*Channa gachua*	n/a	ACS3403	no		10
73	*Channa gachua*	n/a	clade 24	no		10
74	*Channa gachua*	n/a	ABV9996	yes	9	10
75	*Channa gachua*	n/a	clade 25	no		10
76	*Channa gachua*	n/a	ACQ3951	no		10
77	*Channa gachua*	n/a	ACI8494	no		10
78	*Channa gachua*	n/a	ABV9969	yes	9	10
79	*Channa gachua*	n/a	ABV9993	yes	9	10
80	*Channa gachua*	n/a	ABV9994	yes	9	10
81	*Channa gachua*	n/a	clade 26	no		10
82	*Channa gachua*	n/a	clade 27	no		10
83	*Channa gachua*	n/a	ACP4442	no		10
84	*Channa gachua*	n/a	clade 28	no		10
85	*Channa gachua*	*Channa stewartii*	ACS3540	no		10
86	*Channa gachua*	n/a	AAC3925	yes	9	10
87	*Channa gachua*	n/a	clade 29	no		10
88	*Channa gachua*	n/a	ACM5826	no		10
89	*Channa gachua*	n/a	AAC3927	yes	9	10
90	*Channa harcourtbutleri*	*Channa gachua*	AAC3926	yes	9	
n/a	*Parachanna insignis*	n/a	ACE8403^(b)^	yes	1	
n/a	*Rasbora trilineata*	*Channa* sp.	ABW0050^(c)^	yes		

^(a)^wrong species assignment in BOLD and GenBank; not used by Serrao *et al*. [32]

^(b)^reassigned by BOLD to BIN ABW0157.

^(c)^not used in this study.

### Detection of misidentified snakehead specimens in GenBank

In several cases we found potentially misidentified and incompletely identified channid specimens in GenBank (Table 1), some of them generated and/or used by [32]. Overall we identified 71 (16.3%) out of the 434 snakehead sequences downloaded from GenBank (not including *C*. sp. KJ937355 that was not used for the final analysis) as potential misidentifications (Table 1). Among these potentially misidentified sequences were 32 (12.9%) out of the 250 previously deposited coxI sequences, which were used by [32], and nine (7.4%) out of the 121 sequences, which were newly generated by [32]. For example there were several issues with samples of the genus *Parachanna* and we found, that none of the *Pa*. *africana* coxI sequences deposited in GenBank are correctly identified (Table 1, S1 Fig). To better understand the cause of confusion in this genus we downloaded all *Parachanna* coxI sequences from BOLD (date of download April 12, 2016) and were able to include 12 new sequences that were released after our initial download of channid coxI sequences from GenBank (March 31, 2015). We also included five *Protopterus annectens* coxI sequences (accession numbers HQ927824 and HM882951-HM882954 belonging to BIN AAL6055), that actually represent *Pa*. *africana* sequences (see below), aligned them and conducted a NJ analysis. The resulting NJ tree is shown in S6 Fig. Based on this result we identified a potentially new *Parachanna* species (*Pa*. sp. DRCongo, BIN AAF7843; see Discussion) with sequences from individuals previously identified as *Pa*. *obscura* (accession numbers HM880234 and KJ937453) or *Pa*. *africana* (accesssion numbers KJ937418, KJ937351, and KJ937391). In addition two *Pa*. *obscura* are wrongly identified as *Pa*. *insignis* (accession numbers AP006042 and NC_022480). Within one of the *C*. *striata* clades (BIN ACB7973) five sequences labelled as *C*. *marulius* (GenBAnk accession numbers KF430019 and FJ459472- FJ459475) were resolved.

Several sequences labelled as *C*. *orientalis* were placed (see Table 1 and Discussion) within several Indian *C*. *gachua* clades (BINs ABV9995, ACA9095, AAC6050, ABA8489 and ACH0185). Five sequences labelled as *C*. *barca* (BIN ACB7513, accession numbers HM117177- HM117181) clustered with *C*. *bleheri* (BIN ACB7513) and another five *C*. *barca* labelled sequences, generated by the barcoding study of [32] (KJ847147- KJ847151), clustered with *C*. *stewartii* (BIN AAF3764). And finally, sequences labelled as *C*. *stewartii* (accession numbers KJ847152- KJ847156) clustered with *C*. *gachua* (BIN ACS3540) and one *C*. *gachua* labelled sequence (accession number KJ937367) was nested among *C*. *harcourtbutleri* (BIN AAC3926) samples.

### Intraspecific divergence, BIN assignment, species delimitation and barcode gap

Several channid species are characterized by deep intraspecific divergences and are split into multiple lineages or BINs (Fig 1) suggesting the presence of additional species diversity, as previously shown by [32]. [32] identified deep "intraspecific" diversity in nine channid species with multiple BINs per "species" (Table 2): *C*. *asiatica*, *C*. *gachua*, *C*. *lucius*, *C*. *marulius*, *C*. *orientalis*, *C*. *punctata*, *C*. *stewartii*, *C*. *striata*, and *Pa*. *insignis*. Note that all the sequences labelled *C*. *orientalis* by [32] are in fact sequences of misidentified *C*. *gachua*; (Table 1) and that [32] assigned two BINs to *Pa*. *insignis* but that BIN ACE8403 has been reassigned by BOLD to BIN ABW0157 (see below) and hence does not no longer count as case of a species with multiple BINs. In our study, we recovered several more cases of deep intraspecific divergence (Fig 1, Table 2) including additional BINs in species already suggested by [32] to harbour high "intraspecific" diversity (e.g. *C*. *gachua*, C. *marulius*, *C*. *lucius*). More importantly, however, we identified several additional species characterized by the presences of multiple BINs (e.g. *C*. *bankanensis*, *C*. *bleheri*, *C*. *micropeltes*, *C*. *ornatipinnis*; Table 2).

**Table 2:** Channid misidentifications. Summary of channid misidentifications and incompletely identified channid specimens with their BIN assignment, GenBank accession numbers and number of specimens found in GenBank, with how many specimens have been used and / or generated by [32].

Speciemen ID wrong	Specimen ID correct	Category	BOLD:BIN	GenBank accession numbers	Information sequences^(a)^	Comment Fig 1
*Channa barca*	*Channa bleheri*	misidentified	ACB7513	HM117177-HM117181	5/5/0	q
*Channa barca*	*Channa stewartii*	misidentified	AAF3764	KJ847147-KJ847151	5/0/0	t
*Channa* cf. *marulius*	Channa marulioides	incomplete ID	AAC6049	KJ937378	1/1/1	f
*Channa* cf. *melanostigmaa*	*Channa melanostigma*?	incomplete ID	ACH1447	KF511545	1/0/0	n/a
*Channa* cf. *nox*	Channa asiatica?	incomplete ID	ACH5881	LR1804	1/0/0	i
*Channa* cf. *stewartii*	*Channa* sp. Bhutan foothills	incomplete ID	AAC6053	KJ937384	1/1/1	s
*Channa gachua*	*Channa andrao*	misidentified	AAC3928	EU342197-EU342198, KJ937393	3/2/1	v
*Channa gachua*	*Channa andrao*	misidentified	ACB8348	HM117187-HM117191	5/5/0	u
*Channa gachua*	*Channa harcourtbutleri*	misidentified	AAC3926	KJ937367	1/1/1	z
*Channa marulius*	*Channa striata*	misidentified	ACB7973	KF430019, FJ459472-FJ459475	5/0/0	j
*Channa orientalis*	*Channa gachua*	misidentified	ABV9995	KJ937374	1/1/1	k
*Channa orientalis*	*Channa gachua*	misidentified	ACA9095	JN245991, JX105470, JX105472-JX105474	5/5/0	m
*Channa orientalis*	*Channa gachua*	misidentified	AAC6050	FJ459480-FJ459484, KJ937436	6/6/1	n
*Channa orientalis*	*Channa gachua*	misidentified	ABA8489	JQ667514, JX983245-JX983249	6/1/0	o
*Channa orientalis*	*Channa gachua*	misidentified	ACH0185	KF742420, KF742438, KJ847117-KJ847131	17/0/0	p
*Channa* sp.	*Channa maculate*	incomplete ID	ABW0048	KJ937350, KJ937357, KJ937398, KJ937405-KJ937406, KJ937439, KJ937447, KJ937452, KJ937454	9/9/9	e
*Channa* sp. ^(b)^	*Rasbora trilineata*	misidentified	BW0050	KJ937355	1/1/1	n/a
*Channa stewartii*	*Channa* sp. Bhutan foothills	incomplete ID	ACH0210	KF742419	1/0/0	r
*Channa stewartii*	*Channa gachua*	misidentified	ACS3540	KJ847152-KJ847156	5/0/0	x
*Parachanna africana*	*Parachanna* sp. DRCongo	misidentified	AAF7843	KJ937351, KJ937391, KJ937418	3/3/3	b
*Parachanna insignis*	*Parachanna obscura*	misidentified	AAF7842	AP006042, NC_022480	2/0/0	d
*Parachanna obscura*	*Parachanna* sp. DRCongo	misidentified	AAF7843	HM880234, KJ937453	2/1/1	b
*Parachanna* sp.	*Parachanna insignis*	incomplete ID	ABW0157	KJ937414	1/1/1	c
*Protopterus annectens*^(b)^	*Parachanna africana*	misidentified	AAL6055	HM882951-HM882954, HQ927824	5/0/0	a

^(a)^number of specimens / used by [32] / generated by [32].

^(b)^not used in this study, only used in S6 Fig.

From the 49 channid BINs originally reported by [32] BIN ACE8403 (represented by *Parachanna insignis*, accession number KJ937414 in [32], their Fig 1) is now reassigned by BOLD to the existing neighbouring BIN ABW0157. In addition, a total of seven new BINs (ABA8625, ACH0185, ACH1447, ACS3403, ACS3540, ACS5422, ACS6326) are reported in BOLD resulting in a total of 55 public channid BINs. However, during our identification searches we also found an additional six "non-public" channid BINs (ACH0210, ACI8494, ACM5826, ACP4442, ACQ3951, ACX6936). In addition, as we showed above, *Channa* sp. (accession number KJ937355) assigned to BIN ABW0050 is actually not a channid, but a danionine cyprinid. Finally, coxI sequences of our three *Pa*. *africana* individuals (LR0166, LR2276, LR2297) resulted in a 100%-99.84% match in BOLD with *Protopterus annectens* (BIN AAL6055), an African lungfish represented by five specimens (accession numbers HM882951-HM882954 and HQ927824). Therefore, BIN AAL6055 is actually a channid BIN not a lungfish BIN. Hence, the total number of snakehead BINs currently in BOLD is 61. Some of our 777 snakehead coxI sequences could be assigned to one of these 61 existing snakehead BINs (see above, S1 Table). However, several individuals in our study could not be included among existing BINs and were assigned to 29 distinct haplogroups based on the NJ analysis (clades 1–29 in Fig 1) and represent potentially new BINs/haplogroups. This raises the total number of snakehead BINs and potential BINs to 90 (Fig 1; S4 Fig; S1 Table) in contrast to the 49 discrete haplogroups or BINs recovered by [32].

**Fig 3:**
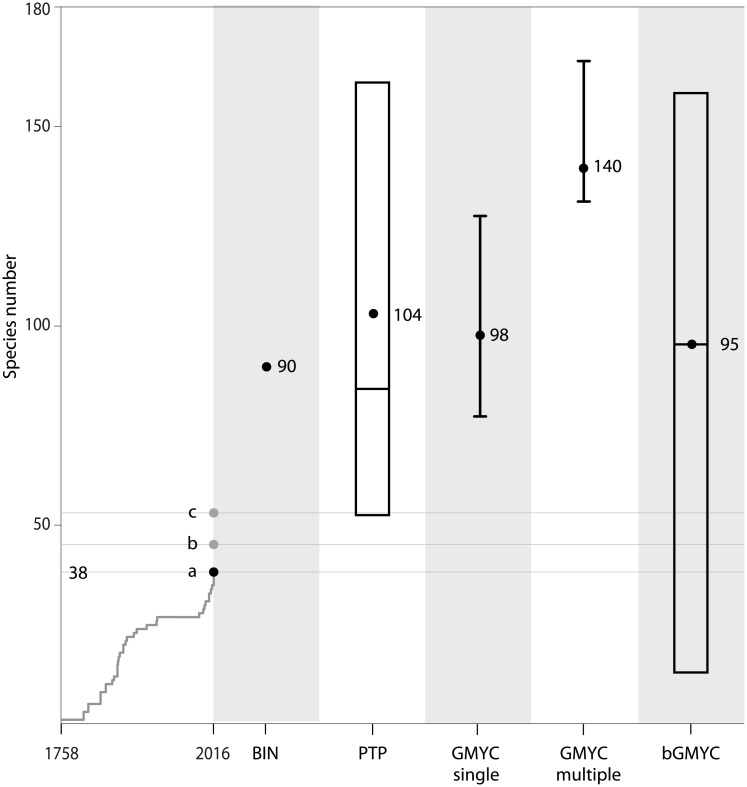
Summary of species delimitation. Cumulative number of channid species from the year 1758 to 2016 and results of channid species numbers estimated by different species delimitation methods. For the PTP analyses the species number is given (dot) and the mean and minimum and maximum range is given based on the analyses of 100 ML bootstrap trees. GMYCs and GMYCm the number of species and the confidence interval are given. For the bGMYC analyses, the mean and minimum and maximum range is given. The horizontal lines indicate different species counts: a) 38 valid species, b) plus seven undescribed species included in this study (see Fig 1): *Channa* sp. Assam, *C*. sp. Bhutan foothills, *C*. sp. Rakhine Yoma, *Channa* sp. Northeast India, *Channa* sp. Tenasserim, *Channa* sp. Mogaung and *P*. sp DRCongo c) plus an additional eight potential species based on a conserved estimate of additional intraspecific diversity within *C*. *bankanensis* (two clades in total), *C*. *marulius* (three clades in total), *C*. *striata* (four clades in total), *C*. *gachua* (at least three clades), totaling a conservative estimate of 53 channid species.

Molecular species delimitation methods gave largely congruent results in suggesting higher species diversity among channids than previously thought. The mean value of delineated species calculated by the tree-based methods varied from 95 (bGMYC, S5 Fig) to 140 (GMYC multiple-threshold). The methods GMYC single-threshold and PTP, recovered 98 and 104 potential species, respectively. With the exception of the GMYC multiple-threshold method, these results are comparable to those obtained by the BIN analysis. Fig 3 summarizes the results from the different species delimitation methods in channids and S4 Fig shows the results of the species delimitation with taxon labels. The PTP Bayesian analysis (bPTP) failed to reach suitable levels of convergence with MCMC 500.000 generations and was therefore not taken into account for the comparison of methods.

**Fig 4:**
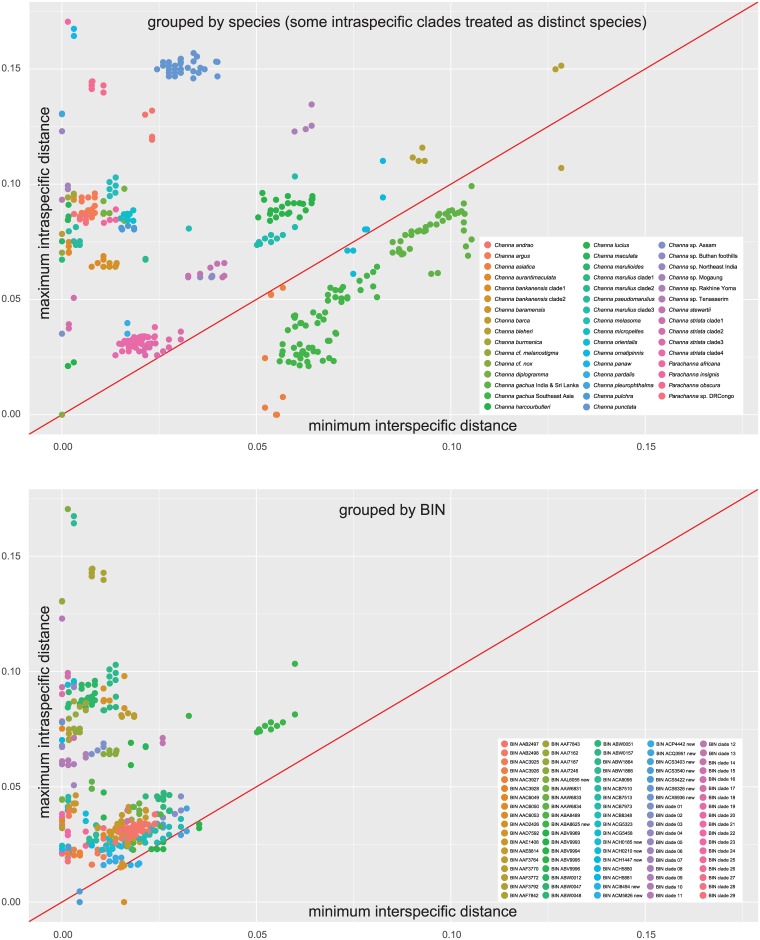
Barcode gap analysis. Dotplot illustrating the presence/absence of local barcode gaps. For each individual the maximum intraspecific distance is plotted against the minimum interspecific distance. The "species" groupings were A) current taxonomy with the exception of species with prominent intraspecific clades that were split into distinct units (*Channa bankanensis*, *C*. *gachua*, *C*. *marulius*, *C*. *striata*), B) according to the 90 BINs identified in this study. The slope 1:1 is indicated by a red line.

To visualize the presence/absence of local barcoding gaps we plotted for each individual the distance to the furthest conspecific against the distance to the nearest non-conspecific. When grouped by traditional species assignment, including some intraspecific clades that were treated as distinct species as in the case of *C*. *bankanensis*, *C*. *gachua*, *C*. *marulius* and *C*. *striata*, the dotplot showed a substantial level of absence of a barcoding gap. The dotplot with individuals grouped to species based on their BIN assignment, on the other hand showed only few instances that did not conform to the presence of a barcoding gap (Fig 4).

## Discussion

### Species misidentifications and perpetuated taxonomic confusions in snakeheads

The main objective of Serrao and co-workers [32] was "to assemble a library of DNA barcode sequences derived from expert identified reference specimens in order to determine the identity and aid invasion pathway analysis of the non-indigenous species found in North America using DNA barcodes". However, our results contradict those of [32] highlighting several problems regarding the identity of some of the material used by them. We are surprised by the large number of misidentified channid coxI sequences in Genbank, some uncritically used by [32] and some even generated by them towards the assembly of a snakehead barcode reference library (Table 2). This is in stark contrast to their stated major goal ([32]:p 3)—“to extend the library of DNA barcode sequences derived from expert-identified reference specimens.” Although [32] mention expert-identified specimens six times in their study, unfortunately, their paper does not include any information on the identity of these taxonomic experts. The most severe case of misidentification we encountered in their dataset involved a case of a sample mix up in which a coxI sequence of the danionine cyprinid genus *Rasbora* had been used in their study as *Channa* sp. (KJ937355; Table 1). Visual inspection of the alignment as well as its position in the NJ tree in Serrao *et al*. [32] (their Fig 1) should have raised alarm bells and a simple BLAST search would have uncovered the "true" identity of this sample. Similarly, the five misidentified African lungfish *Protopterus annectens* from the barcoding study of [49] that are in fact *Parachanna africana* (S6 Fig) could have been easily discovered through a more critical examination of their NJ tree ([49], their Fig 1). There, the five "*Protopterus annectens"* clustered with *Parachanna obscura* while four individuals of *Protopterus* sp. were resolved in a very different position. Clearly, basic quality controls such as automated BLAST searches and more careful examinations of distance trees based on coxI barcodes are needed to avoid such issues caused by sample misidentifications or sample mix-ups. By far the largest number of misidentifications (35 out of 71, Table 2) involved individuals of *Channa gachua* from India that were misidentified as *C*. *orientalis*. *Channa orientalis*, a species without pelvic fins, is restricted to the island of Sri Lanka [50] but this name has been repeatedly used erroneously in the Indian ichthyological literature (e.g. [51, 52, 53]) up to the present day for *C*. *gachua*, a species with pelvic fins. As pointed out previously [5] barcoding initiatives are only successful if the barcoded taxa have been properly identified and thus the study by Serrao *et al*. [32] has increased confusion about channid taxonomy rather than removing or at least reducing it. We hope that our study will help resolving perpetuated taxonomic confusions in snakeheads by providing a clean slate and that it will serve as a reference point for future molecular systematic and DNA barcode studies of this interesting fish group.

### Underappreciated snakehead diversity- the effects of historic over-lumping

Although only 38 channid species are currently being considered valid, over 90 species-level names are available. This large proportion of non-valid snakehead names can partly be explained by their confusing taxonomic history that is characterized by alternating periods of over-splitting and over-lumping. The over-lumping frequently involved the unjustified synonymizing of allopatric sister species. Multiple BINs were assigned to several species in the study of [32] and we found several additional cases of underappreciated diversity mainly in the species *C*. *bankanensis*, *C*. *gachua*, *C*. *marulius*, *C*. *striata* (Table 1). It is important to note that different deeply split lineages within a species complex tend to show geographic separation. Our extensive barcoding study recovered hitherto unknown "intraspecific" diversity in a total of 15 channid species (Table 1) and hence supports previous hypotheses that some current species-level taxa in the genus *Channa* actually represent species complexes and not individual species [32]. For example Britz *et al*. [48] showed that the previously synonymized *C*. *pseudomarulius* is a valid species.

Not unexpected is the result that the lineage currently referred to as *C*. *gachua* is a confusingly difficult species-complex, with two widespread lineages that do not even seem to be closely phylogenetically associated with each other. One western lineage (lineage 1 in Fig 1), which includes the true *C*. *gachua*, is restricted to the area west of the Indo-Burman ranges (i.e. Rakhine Yoma and Chin Hills) and covers at least Sri Lanka, India, Nepal, Bangladesh, and the Rakhine area of Myanmar, showing a high level of divergence among the different samples with a maximum p-distance of 10.53% between these different groups. The base of this lineage is made up by several specimens originating from the Western Ghats area of peninsular India and from Sri Lanka; the latter including the pelvic-fin less species *C*. *orientalis*, which is restricted to Sri Lanka and the taxon referred to as *C*. *gachua* from Sri Lanka, for which the name *C*. *kelaartii* is available. Diversity in this part of the tree is much higher than previously expected and even the pelvic-fin less *C*. *orientalis* is separated into two distinct lineages with a minimum sequence difference of 7.33% (p-distance).

An analogous situation applies to *Channa gachua* from east of the Indo-Burman ranges from Myanmar reaching east to Vietnam and southern China and south to Indonesia and Malaysia (lineage 2 in Fig 1). Genetically surprisingly different from members of the *C*. *gachua* species complex west of the Indo-Burman ranges with a maximum sequence divergence of 8.10% (p-distance) within lineage 2, the eastern lineage also shows a level of intra-complex diversity that is concomitant with the wide distributional range of the group. Although several names are available we suggest referring to this lineage as *C*. *limbata*, the oldest available name, until further detailed studies have reliably identified additional subunits in this eastern lineage. Unexpectedly, two specimens from southern Peninsular India (ChCh1 and Chkk), which we anticipated to group with other *C*. *gachua* specimens of the western lineage (lineage 1 in Fig 1), show greater sequence similarity with specimens in the *C*. *limbata* complex and are recovered in the middle of this eastern lineage. Both the western and eastern lineage of what has been called *C*. *gachua* to date show only limited morphological differences when only preserved specimens are studied. As in the case of the numerous species of the labyrinth fish genus *Betta (e*.*g*. [54]), including colour pattern information from live specimens, especially males in breeding condition, may help distinguishing taxonomic groups within the western (*Channa gachua*) and eastern (*C*. *limbata*) lineages.

One species complex is the taxon called *C*. *striata* in recent literature. Very widely distributed in Asia from Pakistan, India and Sri Lanka across Myanmar, Thailand, Cambodia and Vietnam in the east to Malaysia and Indonesia in the south, *C*. *striata* is a species complex with deep intraspecific divergences between samples (Fig 1), clearly identifying the need for further detailed morphological and accompanying genetic analyses to resolve the species-level units in this group reliably (see also [55]). Interestingly samples of *C*. *striata*, the type locality of which is in Tranquebar on the southeastern coast of India, showed very low levels of genetic divergence across the Indian subcontinent. We have identified several additional examples of deep genetic splits in putative species complexes highlighting underappreciated species diversity briefly discussed here.

*Channa lucius* with its type locality in Java has a wide distributional range occurring along the Tenasserim mountain range in Myanmar east to the Mekong and south to the Sunda islands. Even though we did not have samples from the entire distributional range of *C*. *lucius*, our analysis identified several deep splits within the species, which follow more or less a biogeographical pattern. Samples from Sarawak group with those from Kalimantan. Another group unites the samples from Peninsular Malaysia and Sumatra, which are widely separated from two samples from Khao Sok in Thailand, a locality still south of the Isthmus of Kra, which one would expect to group with the Peninsular Malaysian samples, as part of the same biogeographic realm. The fourth grouping gathers samples from different areas of northern, eastern and southern Borneo, including one sample from Bangkok. Additional samples from the entire range of *C*. *lucius* are necessary to cover the wide area of distribution and to be able to better understand any biogeographically significant units. Meristic and morphometric characters of the different populations will also need to be studied comparatively to demonstrate whether the genetically identified units within *C*. *lucius* may have correlated differences in morphological characters. Detailed studies looking at morphological variation within the *C*. *lucius* complex are necessary, which will receive little help from the study of colour pattern variation, in this camouflaging species with mostly black, brown and white colours.

Described as early as 1758 by Linnaeus, *C*. *asiatica* is the first scientifically known snakehead species. Our results show two widely separated lineages, even though the samples with known locality information originated from the same province in China, Guangdong.

*Channa ornatipinnis* was originally collected from a small stream on the eastern slope of the Rakhine mountain range draining east into the Ayeyarwaddy. Our analysis has identified three separate surprisingly different units among *C*. *ornatipinnis*. The specimens from the type locality form the closest relatives of another group, which consists of specimens from another Ayeyarwaddy tributary about 70 km southeast of the type locality. The specimens from there differ in colour pattern from those of the type locality and the genetic difference further confirms their separate status. The samples of the third unit within *C*. *ornatipinnis* originated from India. The significant differences in coxI nucleotide sequences call for a detailed study of the taxonomic status of the *C*. *ornatipinnis* material other than that from the type locality.

Two separate units of *Channa bleheri* can be distinguished in our analysis. All specimens are from the aquarium trade and without precise locality information, except for one, which was collected near Dibrugarh, Assam in North East India. The type locality was given in the original description as “Upper Dibru at Guijan” in Assam, and the species has been recorded from the Dikrong river in Arunachal Pradesh and the Tinsukia district near Dibrugarh in Assam. The other unit consists of two specimens reportedly collected from northern West Bengal, more than 600 km further west. The deep divergence between the two samples highlights the need for a thorough morphological study of material of both lineages.

### Undescribed diversity or how many species of snakeheads are there?

The results of the current study highlight unexpected and yet undescribed diversity in the genus *Channa*: *C*. sp. Assam, *C*. sp. Bhutan foothills, *C*. sp. Rakhine Yoma, *C*. sp. Northeast India, *C*. sp. Tenasserim, *C*. sp. Mogaung. All of these six undescribed species are found in the Eastern Himalayan biodiversity hotspot (EHH), which includes the southern foothills of the Eastern ranges of the Himalayas, the Indo-Burman Ranges as well as the elevated Shillong-Mikir Hills Plateau that is surrounded by the Assam valley and the Bengal basin planes. The EHH plays a vital role in snakehead diversity harbouring several narrow range endemics, all members of the *Channa gachua* group. Eight out of the ten snakehead species described in the last 25 years originated from either NE India or N Myanmar and thus were located in the EHH and it is expected that over the next few years more snakehead species will be discovered from this region many of which show large differences in coloration rather than morphology among each other.

Unexpectedly, we also encountered previously unrecognized diversity in the genus *Parachanna* (*Pa*. sp DRCongo) demonstrating the presence of four distinct clades, in which currently only three species are recognized: *Pa*. *obscurus*, *Pa*. *africana*, and *Pa*. *insignis*. While *Pa*. *africana* is a distinctly coloured and easily recognizable species, *Pa*. *obscurus* and *Pa*. *insignis* have been repeatedly confused in the literature and even been considered synonyms [56]. We have been able to include *Pa*. *obscura* samples from a range of localities including different river basins in West Africa and the Nile basin. Despite the distance of more than 3000 km between some of the sampling localities, genetic diversity among the *Pa*. *obscura* samples is surprisingly low (maximum p-distances within BIN AAF7842 is 1.07%). *Parachanna insignis* was originally described form the Ogoué in Gabon, but is widely distributed in the Congo basin [57]. In addition to these three species our results identified a fourth group based on samples that were either misidentified as *Pa*. *africana* or *Pa*. *obscura* and were assigned to BIN AAF7843. The range of p-distances between this group of what we call here *Pa*. sp. DRCongo and *Pa*. *insignis* (BIN ABW0157), its sistergroup, was 8.32–9.37%. *Parachanna* sp. DRCongo has thus far been recorded from the Congo river and its tributaries between Kisangani and Kinshasa. They likely represent an undescribed species of African snakehead, which occurs sympatrically with *Pa*. *insignis*. It is conceivable that these samples match records from the Congo river listed under *Pa*. *obscura* by [56] and [57]. *Parachanna obscura* is a species otherwise restricted to the Nilo-Sudan ichthyofaunal province (sensu [58]) in Africa (see [57]) and its occurrence in the Congo basin can be considered unusual for a fish species of that distributional pattern.

There is a large discrepancy between the currently recognized species diversity in snakeheads and estimates based upon single locus species delimitation approaches. While there are currently only 38 valid channid species the inclusion of seven undescribed species (i.e. *C*. sp. Assam, *C*. sp. Bhutan foothills, *C*. sp. Rakhine Yoma *Channa* sp. Northeast India, *C*. sp. Tenasserim, *C*. sp. Mogaung and *Pa*. sp DRCongo) and the additional eight putative species within the *C*. *bankanensis*, *C*. *gachua*, *C*. *marulius*, and *C*. *striata* species groups (see above) would conservatively raise the number of channid species to 53 (Fig 1). This is still a much smaller estimate than those obtained with the different species delimitation methods employed for this study indicating mean values ranging from 84 to 124. While several of the delimitated lineages are consistent across the different methods and hence should provide us with a conservative estimate of species boundaries, incongruence across methods could either point to differences in the power to detect cryptic lineages or could indicate that method assumptions in one or more of the methods have been violated [6]. Clearly, single-locus mtDNA species delimitation approaches only provide putative species, or operational taxonomic units (OTUs; (11]). Further integrative approaches are obviously needed for providing a better taxonomic understanding of snakehead diversity, including new species descriptions and taxonomic revisions of the group.

## Conclusions

By incorporating 343 novel snakehead coxI sequences from specimens determined by taxonomic experts of the group, and combining them with an additional 434 coxI sequences from GenBank we were able to highlight several problems with previous efforts towards the assembly of a snakehead reference barcode library. We identified several instances of species misidentifications but with the inclusion of our own data were able to solve these cases of perpetuated taxonomic confusion. Different species delimitation approaches are congruent in suggesting potentially a much higher species diversity within snakeheads than currently recognized. This higher species diversity is mostly the result of either the incorporation of undescribed narrow range endemics from the Eastern Himalaya biodiversity hotspot or the resolution of several widespread species into geographically well-defined lineages characterized by deep genetic splits between each other. In the latter case, over-lumping in the past has deflated the actual species numbers and available names exist for many of these clades, which need to be revised by *Channa* taxonomists. However, in most cases there is clearly an urgent need for future morphological work, especially for the *C*. *gachua* species complex to better characterize genetically identified clades.

## Supporting information

S1 Fig Neigbour joining tree based on HKY distance. Misidentified and incomplete identified specimens are indicated with a red dot.
(EPS)

S2 Fig 50% Majority rule consensus tree of the neighbour joining tree based on HKY distances. Bootstrap values from 1000 pseudoreplicates are shown.
(EPS)

S3 Fig Maximum likelihood tree of the 423 taxa data set that only included unique haplotypes. Bootstrap values from 1000 pseudoreplicates are shown.
(PDF)

S4 Fig Snakehead chronogram from the BEAST analysis using a coalescence prior. Species delimitations based on BIN, GMYC single, GMYC multiple, and PTP thresholds are indicated by black bars. Misidentified and incomplete identified specimens are indicated with a red dot.
(EPS)

S5 Fig Result of the bGMYC analysis showing sequence-by-sequence distribution of posterior probabilities. The coloured table is a matrix with the probabilities of sequences to be conspecific, ranking from yellow to red: the highest to the lowest values. The timetree corresponds to the consensus tree from BEAST analysis.
(EPS)

S6 Fig Neigbour joining tree of *Parachanna* including additional sequences. The lineage corresponding to the new species *Pa*. sp. DRCongo is highlighted in light red.
(PDF)

S1 Table Table of specimens, GenBank accession numbers, BOLD identification, BIN assignment and locality information. (XLSX)
