# Supplementary figures and images for: Barcoding snakeheads (Teleostei, Channidae) revisited: Discovering greater species diversity and resolving perpetuated taxonomic confusions
Source: PLoS One. 2017 Sep 20;12(9):e0184017. doi: 10.1371/journal.pone.0184017 (PMC5606936; doi:10.1371/journal.pone.0184017)

Nandus nandus JQ3713845

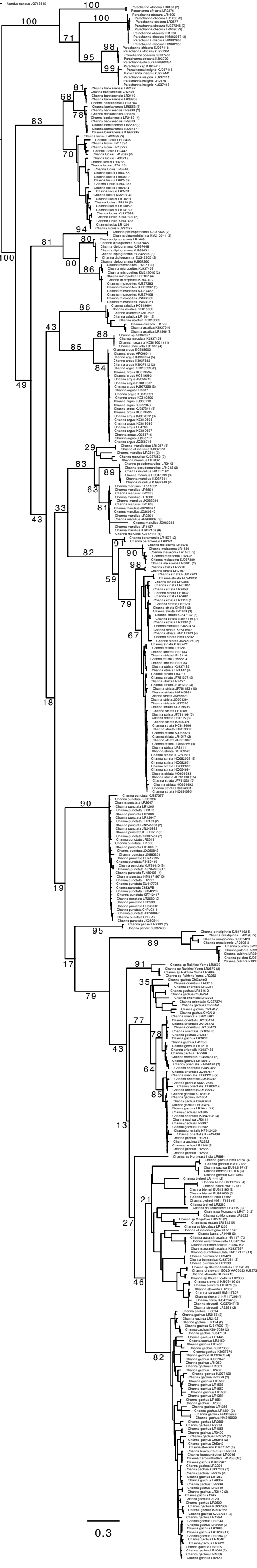

0.3

Supplement: S3 Fig — Bootstrap values from 1000 pseudoreplicates are shown. (PDF) [file pone.0184017.s003.pdf]

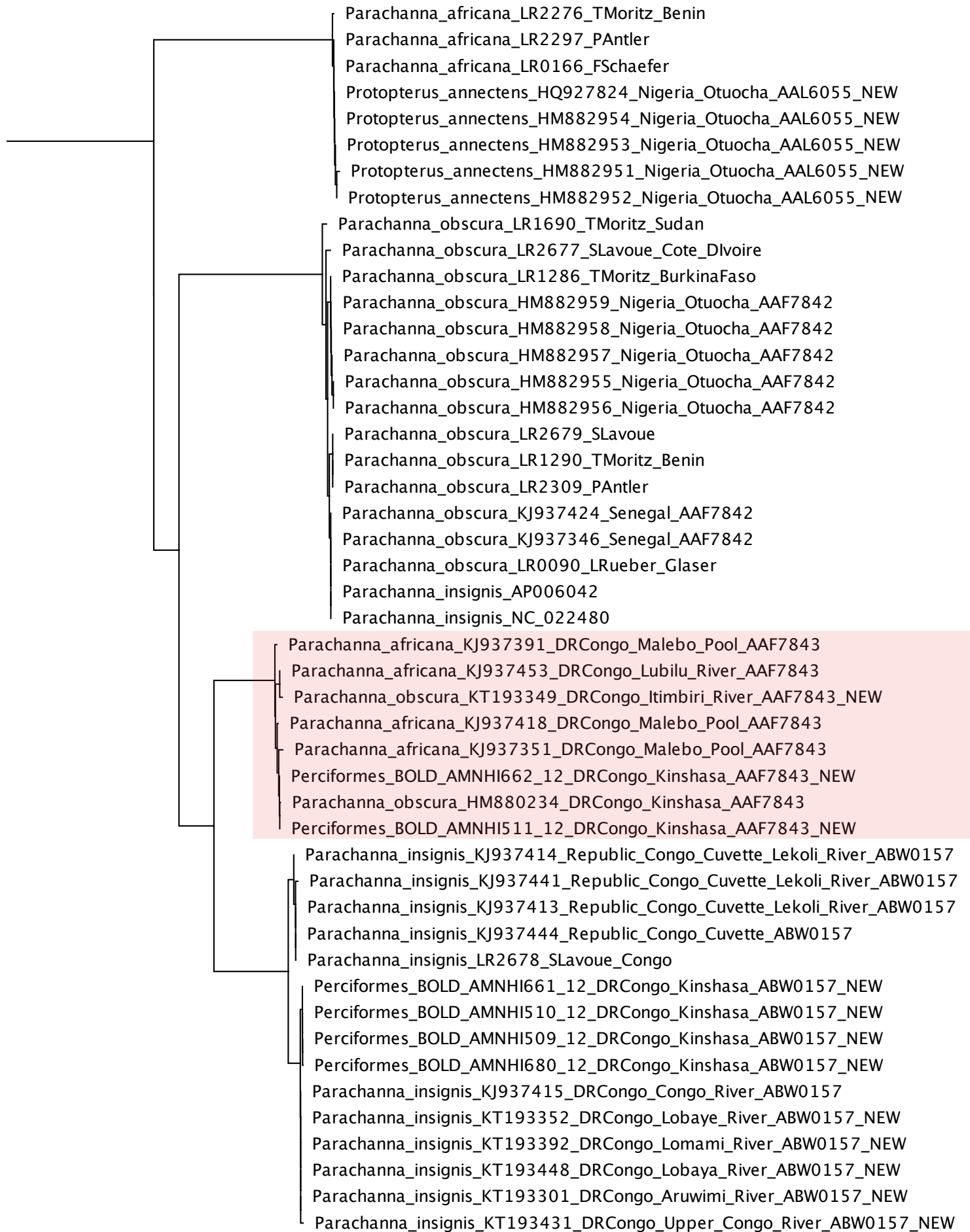

0.07

Supplement: S6 Fig — The lineage corresponding to the new species Pa. sp. DRCongo is highlighted in light red. (PDF) [file pone.0184017.s006.pdf]
